# Physicians’ attitudes towards reproduction in young patients with early breast cancer in China

**DOI:** 10.1007/s10549-020-05854-5

**Published:** 2020-08-10

**Authors:** Yuzhu Zhang, Liping Dian, Xiaoqing Wei, Junyan Huang, Yang Sun, Xue Song, Chunmin Yang, Mengling Kang, Aihua Ou, Qianjun Chen, Rui Xu

**Affiliations:** 1 Breast Department, Guangdong Provincial Hospital of Chinese Medicine, Guangzhou, China; 2 The Second Affiliated Hospital of Guangzhou University of Chinese Medicine, Guangzhou, China; 3 The Second Clinical College of Guangzhou University of Chinese Medicine, Guangzhou, China

**Keywords:** Attitudes, Physicians, Reproduction, Breast cancer, Young patients

## Abstract

**Background:**

As more young patients with breast cancer undergo treatments and obtain good prognoses, the issue of postoperative reproduction in breast cancer patients has attracted more attention.

**Methods:**

We conducted a prospective, cross-sectional survey of 2000 breast cancer-associated physicians using a 24-items questionnaire adapted from prior guides. Then we used a multivariable linear regression model to confirm independent associations between the propensity of physicians’ attitudes toward reproduction and physicians’ specific demographic characteristics.

**Results:**

A total of 911/1249 (72.93%) eligible physicians completed the questionnaire. Regarding the most concerning topic of whether breast cancer patients could conceive, 65 (7.1%) physicians having low and 457 (50.2%) physicians having high propensity for recommending reproduction. For ductal carcinoma in situ (DCIS) after surgery and radiotherapy, 599 (65.8%) physicians did not agree with the recommendation to conceive. 231 (25.4%) highly agree with the recommendation of reproduction for 2 years after surgery in invasive breast cancer patients with lymph nodes-negative. Only 140 (15.4%) physicians did not agree with the recommendation for 5 years after surgery in invasive breast cancer patients with lymph nodes-positive. A total of 861 (94.5%) physicians stated that they advised the patients to consult experts from other disciplines, such as gynecology, oncology, genetic and psychology disciplines. In multivariable analysis, more positive attitude toward reproduction was significantly associated with male, more than 11 times of participating in academic forum on breast cancer, 1–2 times of consulting about reproduction problems after breast cancer surgery per outpatient service and more than 11 min spending on solving the problem about reproduction in early breast cancer.

**Conclusion:**

This study showed that attitudes towards reproduction of young breast cancer patients from physicians in China. Physicians had a high propensity for recommending reproduction. Compared with the two reproduction guidelines recommendation when to reproduce in different circumstances for breast cancer patients, physicians from China remained a relatively conservative attitude. Most physicians advised the patients to consult experts from other disciplines, such as gynecology, oncology, genetic and psychology disciplines.

## Introduction

Breast cancer has the highest incidence among female malignancies. A total of 0.5–2% patients is diagnosed with breast cancer before age 20, and approximately 20% are diagnosed before age 30 [1]. As more and more young breast cancer patients receive standardized treatments and a good prognosis, young patients’ reproduction has been given more and more attention, especially because of the implementation of the Two-Child Policy in China. Patients with breast cancer are always advised to avoid becoming pregnant or to put off reproduction for at least 2 years after treatment, especially for luminal breast cancer, as reproduction would induce cancer recurrence and worsen survival [2]. However, some retrospective studies have contradicted the hypothesis that reproduction in breast cancer patients is safe and even has established benefits for the prognosis of patients [3–5]. Interestingly, Theriault et al. [4] found a 41% lower risk of death among women who became pregnant after a breast cancer diagnosis compared to women with no reproduction. In addition, Theriault [5] also showed that regardless of estrogen receptor status, there was no difference in disease-free survival (DFS) between women who became pregnant after their breast cancer diagnosis and those who did not, and there was a benefit in overall survival (OS) among those who were pregnant. In fact, the study selection bias and research correlation index still need to be verified. Generally, that reproduction endangers breast cancer patient survival, especially in patients who are estrogen receptor (ER)–positive, remains controversial.

Although most young breast cancer patients still desire to conceive after systemic cancer therapy, the reproduction rate is 70% lower than that of the general population due to the reproductive decline caused by treatment, including gonadotoxic chemotherapy and endocrine therapy [6, 7]. reproduction occurring more than 1 year after the diagnosis of breast cancer did not appear to affect patient survival, and the appropriate time for patients to attempt pregnancies after undergoing breast cancer treatment is still not clear. The 2017 Rehabilitation Therapy Consensus on Breast Cancer in China [8] and the British Royal Society of Obstetrics and Gynecology [9] have given advice about reproduction according to the patients’ clinic condition. This consensus intends to help relevant physicians guide young breast cancer patients with their fertility needs. However, we do not know the attitudes of breast cancer-associated physicians toward the postoperative reproduction of breast cancer patients.

Herein, to examine the attitude of breast cancer-associated physicians regarding reproduction after systemic cancer therapy, we designed a prospective attitude survey with specific clinical scenarios regarding reproduction in young patients with breast cancer according to the guides above. The characteristics of the physicians with a relatively high propensity for recommending reproduction in the specific clinical context were analyzed.

## Methods

### Sampling and data collection

This analysis was derived from a prospective, cross-sectional study about breast cancer-associated physicians’ attitude toward reproduction in early breast cancer patients, which was conducted from January 24, 2019, to June 20, 2019. To ensure that the investigators involved were breast cancer physicians, participants were from the Chinese Society of Clinical Oncology, Committee of Breast Cancer (CSCO-BC). We designed the questionnaire on ‘Wenjuanxing’ (https://www.wjx.cn) and distributed the questionnaire by WeChat.

Before the formal investigation, we conducted extensive pretesting. Then we chose 90 s as a screening criterion because it takes an average of 90 s for a partner of our research team to complete these questionnaires. If it is less than 90 s, it means that he is likely to fill in the answers without fully reading the questions. The questionnaire content of this study mainly included 3 parts: (1) the collection of baseline information, (2) the evaluation of attitudes toward the scenario-based treatment strategy, and (3) attitudes about decision-making and associated risk factors. The study was approved by the ethics committee of GuangDong Provincial Hospital of Chinese Medicine (ZE2019-037).

### Measures

To investigate the physicians’ attitudes toward reproduction in young patients with early breast cancer, we designed 11 clinical scenarios. First, we used 4 questions to ask physicians whether a subsequent reproduction would alter patients’ risk of disease recurrence and whether systemic cancer therapy would affect fetal health. The questions were as follows: (1) Do you think a patient with breast cancer can become pregnant? (2) Do you think reproduction has negative effects on breast cancer patients? (3) Do you think reproduction in early breast cancer patient has negative effects on the fetus? and (4) do you think patients can breastfeed after delivery? Second, we used 4 questions to determine the physicians’ attitudes about the timing of reproduction after breast cancer treatment. The questions were as follows: (1) will you suggest patients with breast cancer become pregnant in situ after surgery and radiotherapy? (2) Will you suggest invasive breast cancer patients who are lymph node-negative become pregnant 2 years after surgery? (3) Will you suggest invasive breast cancer patients who are lymph nodes-positive become pregnant 5 years after surgery? And (4) Will you suggest patients who need endocrine therapy stop endocrine therapy 3 months before reproduction and continue after lactation? Third, we also took into account questions regarding the BRCA1/2 mutation, hormone drugs promoting ovulation and use of a multidisciplinary team (MDT). The questions were as follows: (1) would having a BRCA1/2 mutation patient affect your suggestions? (2) Would you suggest patients use hormone drugs to promote ovulation? (3) Would you agree to an MDT consultation to make a fully informed decision regarding the patients’ reproduction?

**Fig. 1:**
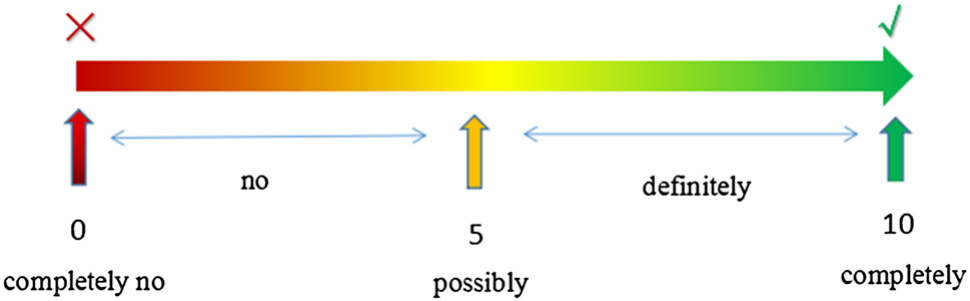
The visual selection diagram of scale scoring indicators in the survey. Responses categories for each issue above are: “0–3” indicates low propensity, “4–6” indicates selective propensity and “7–10” indicates high propensity. The score ranges from 0 to 10, with 0 indicating completely no, 1–3 indicating low propensity (the larger the scale score was in this part, the higher propensity for possibly was with “no add one”, “no add two” “no add three”), 5 indicating possibly; 4–6 indicating selective propensity (possibly minus one, possibly minus add one), 7–9 indicating high propensity with certain influence (the larger the scale score was in this part, the higher propensity for completely yes was with “definitely minus three”, “definitely minus two” “definitely minus one”), and 10 indicating definitely yes

Responses categories for each issue above are as follows: “0–3” indicates low propensity, “4–6” indicates selective propensity, and “7–10” indicates high propensity (Fig. 1). To improve test efficiency and reduce selection bias, item responses were fit by a graded item response model to create a latent scale. Therefore, different propensities of attitudes about physicians recommending breast cancer patients become reproduction are shown according to the scale value of different issues. Other physicians’ measures included the following demographic information: sex, region, years of practice, number of outpatient breast cancer patients, frequency of academic forum participation, frequency of science popularization and propaganda activities about reproduction of early breast cancer, time spent answering issues, and frequency of offering advice to consult with experts in other subjects.

### Statistical analysis

We first described the demographic characteristics of breast cancer-associated physicians and evaluated the reports of physicians recommending options for 11 specific clinical scenarios. Following the development of the 10-point scale for tendency of recommending reproduction, physicians were scored and categorized as having a low (“0–3” indicate low propensity), selective (“4–6” indicate selective propensity) or high propensity (“7–10” indicate high propensity). Next, we used a multivariable linear regression model to confirm independent associations between the propensity of physicians’ attitudes toward reproduction and physicians’ specific demographic characteristics. All analyses were conducted using SPSS, version 23.0. All reported *P* values were 2-sided with a 0.05 significance level.

## Results

**Fig. 2:**
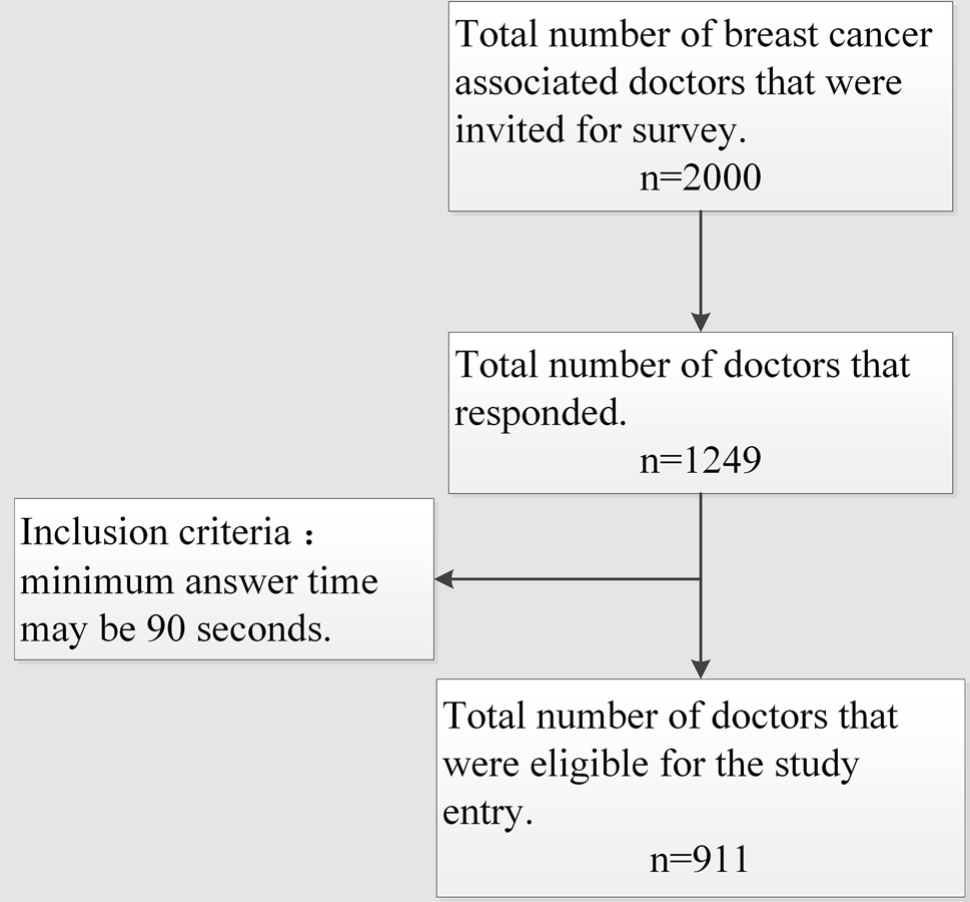
The schematic diagram of investigation process

In total, 2000 breast cancer-associated physicians were invited and 1249 physicians responded (1249/2000, 62.35%), of which 911 (911/1249, 72.93%) were eligible according to the inclusion criteria that the minimum answer time was 90 s to adjust for an assumed time bias. 6 physicians were excluded because they did not want to be surveyed (Fig. 2).

### Characteristics of physicians

Among 911 respondents (Table 1), 479 (52.6%) were female physicians, and only 304 (33.4%) physicians from developed areas. The proportion of physicians with less than 5 years of practice was the largest group (424 physicians, 46.5%) and the vast majority of respondents (756 physicians, 83%) show a volume of fewer than 40 breast cancer patients per outpatient service. A total of 372 (40.8%) physicians expressed that they took part in more than five times academic conference per year. Regarding the popularization of breast cancer patient reproduction, 278 physicians (30.5%) stated that they had never encountered the consultation about fertility with breast cancer patients. As the physicians’ practice years increased, the number of physicians who had participated in academic forums more than 5 times and in popular science activities showed an increasing trend. The time physicians spend answering inquiries was mostly (689, 75.6%) between 2 and 10 min.

**Table 1:** Breast cancer-associated physicians’ sample characteristics (*N* = 911)

Characteristic	Value
Gender, no. (%)	
Female	479 (52.6)
Male	432 (47.4)
Come from which area, no. (%)	
Developed area	304 (33.4)
Underdeveloped area	607 (66.6)
Years of practice, no. (%)	
< 5 years	424 (46.5)
5 years ~	285 (31.3)
10 years ~	202 (22.2)
Volume of breast cancer patients per outpatient service, no. (%)	
< 20	431 (47.3)
20 ~	325 (35.7)
40 ~	95 (10.4)
> 60	60 (6.6)
Times of participating in academic forum on breast cancer, no. (%)	
< 2	148 (16.2)
2–4	391 (42.9)
5–10	275 (30.2)
≥ 11	97 (10.6)
Times of science popularization about reproduction of early breast cancer, no. (%)	
0	278 (30.5)
1	274 (30.1)
2	185 (20.3)
> 2	174 (19.1)
Times of consulting about reproduction problems after breast cancer surgery per outpatient service, no. (%)	
1–2	445 (48.8)
3–4	274 (30.1)
5–6	93 (10.2)
> 6	99 (10.9)
Time spent solving the problem about reproduction in early breast cancer, no. (%)	
< 2 min	97 (10.6)
2 min ~	429 (47.1)
6 min ~	260 (28.5)
11 min ~	125 (13.7)

Of the initial 2000 breast cancer-associated physicians invited, 1249 physicians responded (62.35% of those were invited) and 911 (72.93% of those responded) were eligible according to the inclusion criteria that the minimum answer time may be 90 s to adjust for guaranteed time bias

### Physicians’ attitude towards reproduction in young patients with early breast cancer

**Fig. 3:**
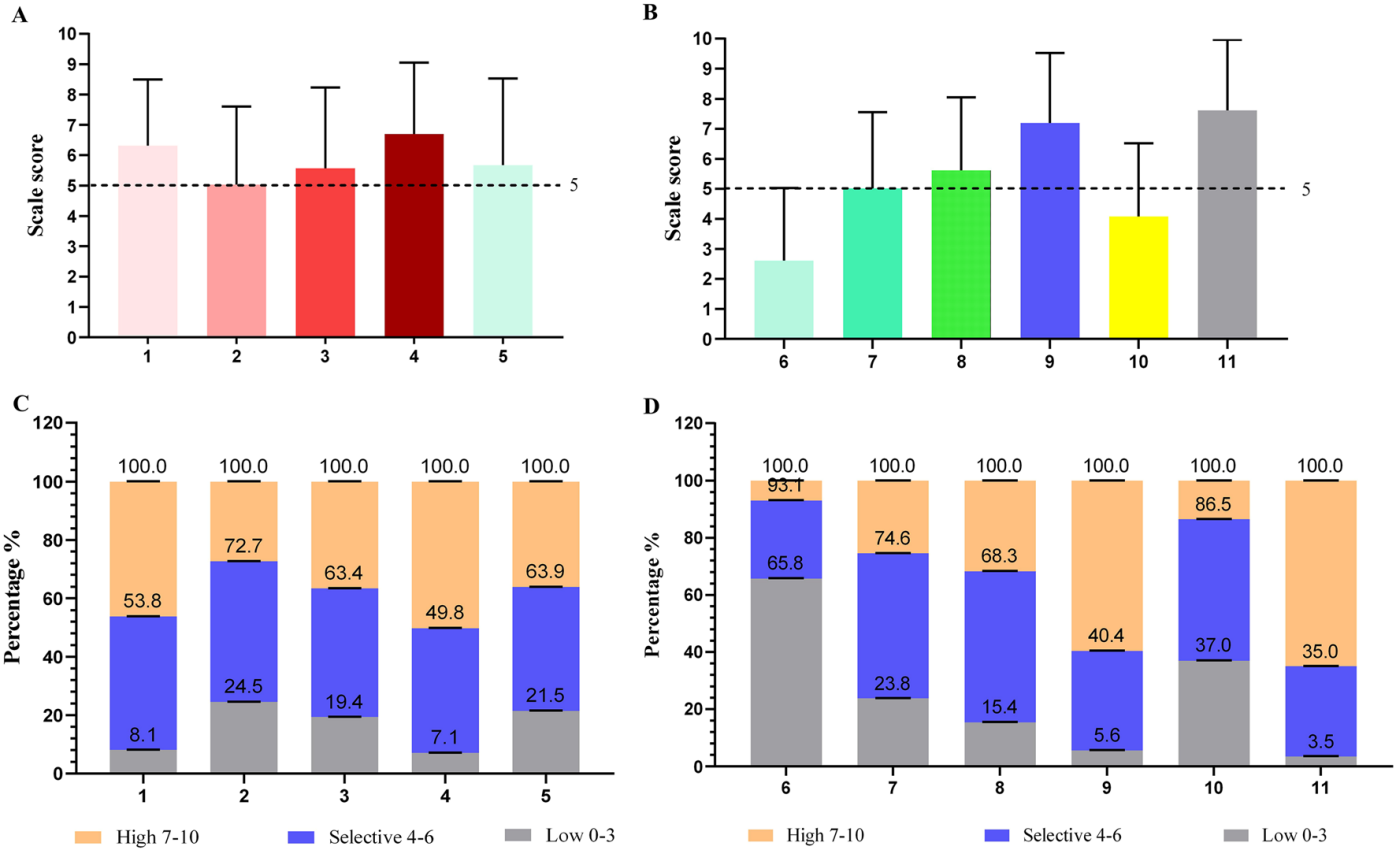
Attitudes towards procreation of young patients with early breast cancer. **a**–**d** 1. Attitudes about pregnancy effects on breast cancer patients; 2. Attitudes about breast cancer patient's pregnancy's effects on the fetus. 3. Attitudes about effects on doctors' decisions because it is a BRCA1-related patient. 4. Attitudes about whether a patient with breast cancer may be pregnant. 5. Attitudes about whether patients may breastfeed after delivery. 6. Attitudes about patients with breast cancer in situ getting pregnant after the surgery and radiotherapy. 7. Attitudes about getting pregnant 2 years after surgery in invasive breast cancer patients with lymph nodes-negative. 8. Attitudes about getting pregnant 5 years after surgery in invasive breast cancer patients with lymph nodes-positive. 9. Attitudes about whether the patients who need endocrinotherapy still need to continue endocrine treatment after lactation. 10. Attitudes about the using of hormone drugs to promote ovulation for patients with breast cancer. 11. Attitudes about agreement for an MDT consultation to make a full decision on the patients’ procreation. Following the development of the 10-point scale of recommended reproductive tendency, doctors were scored and categorized as having low, selective or high propensity (“0–3” indicate low propensity, “4–6” indicate selective propensity and “7–10” indicate high propensity)

As both the health of the mother and fetus need to be considered, 421 (46.2%) and 249 (27.3%) physicians thought reproduction had certain effects on patients and fetuses when breast cancer patients attempted reproduction (Fig. 3a and C *x* = 1 and 2). In addition, in a scenario with BRCA-1/2 mutation patients, 734 (80.6%) physicians would be influenced to help patient of making reproduction decisions (Fig. 3a and c, *x* = 3). Regarding the most concerning topic of whether breast cancer patients could conceive, 65 (7.1%) physicians having low and 457 (50.2%) physicians having high propensity for recommending reproduction (Fig. 3a and c, *x* = 4). Physicians who responded to the issue whether patients may breastfeed after delivery, 196 (21.5%) did not and 329 (36.1%) did recommend breastfeeding (Fig. 3a and c, *x* = 5).

When the breast cancer patients become pregnant in different situation? For ductal carcinoma in situ (DCIS) after surgery and radiotherapy, 599 (65.8%) physicians did not agree with the recommendation compared with 249 (27.3%) who opted for selective recommendation and 63 (6.9%) who highly recommended it (Fig. 3b and d, *x* = 6). For 2 years after surgery in invasive breast cancer patients with lymph nodes-negative, 217 (23.8%) physicians did not agree with the recommendation compared with 463 (50.8%) who opted for selective recommendation and 231 (25.4%) who highly recommended it (Fig. 3b and d, *x* = 7). For 5 years after surgery in invasive breast cancer patients with lymph nodes-positive, 140 (15.4%) physicians did not agree with the recommendation compared with 482 (52.9%) who opted for selective recommendation and 289 (31.7%) who highly recommended it (Fig. 3b and d, *x* = 8). Most physicians (*n* = 860, 94.4%) indicated that patients who needed adjuvant endocrine therapy may continue to receive endocrine therapy after reproduction and lactation (Fig. 3b and d, *x* = 9, Table 5); There were 337 (37.0%) physicians who did not recommend hormone drugs to promote ovulation, compared with 451 (49.5%) who selectively agreed and 123 (13.5%) who highly agreed (Fig. 3b and d, *x* = 10, Table 5). 32 (3.5%) physicians did not agree with having an MDT consultation to make a fully informed decision on the patients’ reproduction status (Fig. 3b and d, *x* = 11, Table 5). A total of 861 (94.5%) physicians stated that they advised the patients to consult experts from other disciplines, such as gynecology, oncology, genetic and psychology disciplines.

### Multivariate analysis of factors that might influence physicians’ attitudes on different issues

We conducted a multivariate analysis of the factors that might influence physicians' attitudes on different issues. We identified several predictors of a more positive attitude toward reproduction (Table 2), including male (OR 1.44 [95% CI, 1.11 to 1.89], *P* = 0.007), more than 11 times of participating in academic forum on breast cancer (OR for less than 2 times: 0.22 [95% CI, 0.12 to 0.41], *P* < 0.001; OR for 2 to 4 times: 0.41 [95% CI, 0.24 to 0.68], *P* = 0.001; OR for 5 to 10 times: 0.54 [95% CI, 0.32 to 0.90], *P* = 0.019), 1–2 times of consulting about reproduction problems after breast cancer surgery per outpatient service (OR 1.74 [95% CI, 1.07 to 2.83], *P* = 0.026) and more than 11 min spending on solving the problem about reproduction in early breast cancer (OR for less than 2 min: 0.63 [95% CI, 0.35 to 1.11], *P* = 0.107; OR for 2 to 6 min: 0.58 [95% CI, 0.38–0.89], *P* = 0.013; OR for 6 to 10 min: 0.88 [95% CI, 0.56 to 1.38], *P* = 0.573).The only variable that was statistically significant among physicians’ attitudes about reproduction effects on breast cancer patients was times of participating in academic forum on breast cancer (*P* = 0.01, Table 3). Physicians who had been in practice for fewer than 5 years (OR 1.74 [95% CI, 1.24–2.46], *P* = 0.001) and more than 6 times of consulting about reproduction problems after breast cancer surgery per outpatient service (OR for 1 to 2 times: 0.45 [95% CI, 0.28 to 0.72], *P* = 0.001; OR for 3 to 4 times: 0.61 [95% CI, 0.38–0.97], *P* = 0.038; OR for 5 to 6 times: 0.91 [95% CI, 0.52–1.58], *P* = 0.736) were more likely to express high propensity attitude on effects of reproduction on fetuses (*P* < 0.01; Table 4). However, there is no significant variable on attitudes about physicians’ decisions for BRCA-1/2 mutation patients (Table 5). While Table 6 shows that five variables were statistically significant among physicians who would and would not consider recommending patients breastfeeding after delivery, including gender, years of practice, times of participating in academic forum on breast cancer, times of science popularization about reproduction of early breast cancer and times of consulting about reproduction problems after breast cancer surgery per outpatient service.

**Table 2:** Multivariate analysis of factors that might influence breast cancer-associated physicians’ attitudes about whether a patient with breast cancer may be pregnant. (*N* = 911)

Factor	Propensity, no			OR (95% CI)	Wald *P* value	*P*
	Low	Selective	High			
Gender						.007
Male	27	166	239	1.44 (1.11–1.89)	.007	
Female	38	223	218	Reference		
Come from which area						.751
Developed area	20	126	158	1.05 (0.79–1.38)	.751	
Underdeveloped area	45	263	299	Reference		
Years of practice						.662
< 5 years	40	190	194	0.95 (0.66–1.37)	.788	
5 years ~	19	116	150	1.10 (0.75–1.60)	.629	
10 years ~	6	83	113	Reference		
Volume of breast cancer patients per outpatient service						.748
< 20	34	170	227	1.06 (0.59–1.92)	.843	
20 ~	22	156	147	0.91 (0.51–1.63)	.748	
40 ~	5	39	51	1.10 (0.56–2.14)	.791	
> 60	4	24	32	Reference		
Times of participating in academic forum on breast cancer						< .001
< 2	20	74	54	0.22 (0.12–0.41)	< .001	
2–4	24	187	180	0.41 (0.24–0.68)	.001	
5–10	16	106	153	0.54 (0.32–0.90)	.019	
≥ 11	5	22	70	Reference		
Times of science popularization about reproduction of early breast cancer						.272
0	17	107	154	1.34 (0.87–2.08)	.184	
1	22	122	130	1.09 (0.72–1.66)	.688	
2	14	90	81	0.90 (0.58–1.40)	.636	
> 2	12	70	92	Reference		
Times of consulting about reproduction problems after breast cancer surgery per outpatient service						< .001
1–2	27	159	259	1.74 (1.07–2.83)	.026	
3–4	23	150	101	0.88 (0.54–1.43)	.594	
5–6	6	37	50	1.50 (0.83–2.69)	.177	
> 6	9	43	47	Reference		
Time spent on solving the problem about reproduction in early breast cancer						.022
< 2 min	11	39	47	0.63 (0.35–1.11)	.107	
2 min ~	35	200	194	0.58 (0.38–0.89)	.013	
6 min ~	9	113	138	0.88 (0.56–1.38)	.573	
11 min ~	10	37	78	Reference

**Table 3:** Multivariate analysis of factors that might influence breast cancer-associated physicians’ attitudes about reproduction effects on breast cancer patients. (*N* = 911)

Factor	Propensity, no			OR (95% CI)	Wald *P* Value	*P*
	Low	Selective	High			
Gender						.132
Male	45	194	193	0.82 (0.63–1.06)	.132	
Female	29	222	228	Reference		
Come from which area						.512
Developed area	24	135	145	1.10 (0.8–1.44)	.512	
Underdeveloped area	50	281	276	Reference		
Years of practice						.482
< 5 years	30	195	199	1.03 (0.73–1.47)	.854	
5 years ~	27	134	124	0.86 (0.60–1.24)	.426	
10 years ~	17	87	98	Reference		
Volume of breast cancer patients per outpatient service						.378
< 20	28	196	207	1.16 (0.65–2.06)	.623	
20 ~	31	154	140	0.90 (0.51–1.59)	.715	
40 ~	13	37	45	0.89 (0.47–1.70)	.723	
> 60	2	29	29	Reference		
Times of participating in academic forum on breast cancer						.010
< 2	13	72	63	0.76 (0.44–1.32)	.332	
2–4	30	199	162	0.85 (0.54–1.36)	.500	
5–10	16	112	147	1.39 (0.87–2.22)	.164	
≥ 11	15	33	49	Reference		
Times of science popularization about reproduction of early breast cancer						.863
0	24	117	137	1.11 (0.73–1.69)	.628	
1	31	114	129	1.05 (0.70–1.58)	.813	
2	9	103	73	0.93 (0.61–1.43)	.749	
> 2	10	82	82	Reference		
Times of consulting about reproduction problems after breast cancer surgery per outpatient service						.389
1–2	49	183	213	0.75 (0.46–1.21)	.233	
3–4	16	146	112	0.76 (0.47–1.23)	.262	
5–6	4	43	46	1.05 (0.59–1.87)	.862	
> 6	5	44	50	Reference		
Time spent on solving the problem about reproduction in early breast cancer						.052
< 2 min	10	38	49	1.03 (0.59–1.78)	.921	
2 min ~	29	196	204	1.01 (0.67–1.52)	.958	
6 min ~	23	134	103	0.66 (0.43–1.02)	.059	
11 min ~	12	48	65	Reference

**Table 4:** Multivariate analysis of factors that might influence breast cancer-associated physicians’ attitudes about breast cancer patient's reproduction's effects on the fetus. (*N* = 911)

Factor	Propensity, no			OR (95% CI)	Wald *P* value	*P*
	Low	Selective	High			
Gender						.181
Male	127	191	114	0.84 (0.65–1.08)	.181	
Female	97	248	134	Reference		
Come from which area						.876
Developed area	71	159	74	1.02 (0.79–1.33)	.876	
Underdeveloped area	153	280	174	Reference		
Years of practice						.004
< 5 years	83	216	125	1.74 (1.24–2.46)	.001	
5 years ~	70	131	84	1.63 (1.15–2.32)	.007	
10 years ~	71	92	39	Reference		
Volume of breast cancer patients per outpatient service						.743
< 20	115	210	106	1.06 (0.61–1.85)	.839	
20 ~	70	157	98	1.23 (0.71–2.13)	.470	
40 ~	26	41	28	1.08 (0.58–2.02)	.810	
> 60	13	31	16	Reference		
Times of participating in academic forum on breast cancer						.854
< 2	29	80	39	1.21 (0.71–2.06)	.482	
2–4	87	204	100	1.03 (0.66–1.62)	.894	
5–10	77	119	79	1.06 (0.67–1.66)	.814	
≥ 11	31	36	30	Reference		
Times of science popularization about reproduction of early breast cancer						.076
0	83	138	57	0.66 (0.44–0.99)	.045	
1	67	131	76	0.89 (0.60–1.32)	.561	
2	39	84	62	1.05 (0.70–1.60)	.807	
> 2	35	86	53	Reference		
Times of consulting about reproduction problems after breast cancer surgery per outpatient service						.001
1–2	143	200	102	0.45 (0.28–0.72)	.001	
3–4	51	149	74	0.61 (0.38–0.97)	.038	
5–6	14	46	33	0.91 (0.52–1.58)	.736	
> 6	16	44	39	Reference		
Time spent on solving the problem about reproduction in early breast cancer						.075
< 2 min	24	46	27	1.18 (0.69–2.01)	.539	
2 min ~	92	219	118	1.16 (0.78–1.71)	.467	
6 min ~	74	120	66	0.77 (0.51–1.17)	.223	
11 min ~	34	54	37	Reference

**Table 5:** Multivariate analysis of factors that might influence breast cancer-associated physicians’ attitudes about effects on physicians' decisions because it is a BRCA-1/2 mutation-related patient. (*N* = 911)

Factor	Propensity, no			OR (95% CI)	Wald *P* value	*P*
	Low	Selective	High			
Gender						.567
Male	100	171	161	0.93 (0.72–1.19)	.567	
Female	77	229	173	Reference		
Come from which area						.605
Developed area	52	143	109	1.07 (0.83–1.39)	.605	
Underdeveloped area	125	257	225	Reference		
Years of practice						.621
< 5 years	66	207	151	1.18 (0.84–1.66)	.330	
5 years ~	57	123	105	1.13 (0.80–1.60)	.495	
10 years ~	54	70	78	Reference		
Volume of breast cancer patients per outpatient service						.500
< 20	84	195	152	1.00 (0.58–1.74)	.998	
20 ~	68	140	117	0.90 (0.52–1.56)	.710	
40 ~	16	37	42	1.27 (0.68–2.37)	.453	
> 60	9	28	23	Reference		
Times of participating in academic forum on breast cancer						.716
< 2	26	75	47	1.09 (0.65–1.85)	.747	
2–4	66	184	141	1.23 (0.79–1.92)	.362	
5–10	53	118	104	1.25 (0.80–1.95)	.332	
≥ 11	32	23	42	Reference		
Times of science popularization about reproduction of early breast cancer						.852
0	66	113	99	0.91 (0.61–1.36)	.633	
1	51	123	100	1.03 (0.69–1.52)	.897	
2	26	93	66	1.06 (0.70–1.59)	.799	
> 2	34	71	69	Reference		
Times of consulting about reproduction problems after breast cancer surgery per outpatient service						.143
1–2	113	172	160	0.62 (0.39–0.98)	.041	
3–4	41	139	94	0.72 (0.45–1.15)	.164	
5–6	8	49	36	0.91 (0.53–1.59)	.750	
> 6	15	40	44	Reference		
Time spent on solving the problem about reproduction in early breast cancer						.568
< 2 min	21	40	36	0.98 (0.58–1.65)	.925	
2 min ~	73	202	154	0.98 (0.66–1.44)	.912	
6 min ~	53	117	90	0.80 (0.53–1.21)	.285	
11 min ~	30	41	54	Reference

**Table 6:** Multivariate analysis of factors that might influence breast cancer-associated physicians’ attitudes about whether patients may breastfeed after delivery. (*N* = 911)

Factor	Propensity, no			OR (95% CI)	Wald *P* value	*P*
	Low	Selective	High			
Gender						.029
Male	89	160	183	1.33 (1.03–1.71)	.029	
Female	107	226	146	Reference		
Come from which area						.297
Developed area	55	134	115	1.15 (0.88–1.50)	.297	
Underdeveloped area	141	252	214	Reference		
Years of practice						< .001
< 5 years	112	198	114	0.52 (0.37–0.74)	< .001	
5 years ~	55	118	112	0.78 (0.55–1.11)	.169	
10 years ~	29	70	103	Reference		
Volume of breast cancer patients per outpatient service						.575
< 20	98	162	171	1.14 (0.65–1.99)	.653	
20 ~	68	160	97	0.92 (0.53–1.61)	.776	
40 ~	20	36	39	1.04 (0.55–1.96)	.904	
> 60	10	28	22	Reference		
Times of participating in academic forum on breast cancer						< .001
< 2	41	73	34	0.26 (0.15–0.45)	< .001	
2–4	88	181	122	0.38 (0.23–0.60)	< .001	
5–10	53	108	114	0.49 (0.31–0.79)	.003	
≥ 11	14	24	59	Reference		
Times of science popularization about reproduction of early breast cancer						.013
0	57	100	121	1.43 (0.95–2.16)	.088	
1	57	117	100	1.25 (0.84–1.85)	.280	
2	45	96	44	0.76 (0.50–1.15)	.195	
> 2	37	73	64	Reference		
Times of consulting about reproduction problems after breast cancer surgery per outpatient service						.040
1–2	88	160	197	1.57 (0.99–2.49)	.058	
3–4	58	153	63	1.07 (0.67–1.71)	.775	
5–6	20	38	35	1.54 (0.89–2.68)	.127	
> 6	30	35	34	Reference		
Time spent on solving the problem about reproduction in early breast cancer						.335
< 2 min	24	44	29	0.90 (0.53–1.54)	.707	
2 min ~	87	194	148	1.10 (0.75–1.64)	.622	
6 min ~	53	107	100	1.33 (0.88–2.02)	.175	
11 min ~	32	41	52	Reference		

On the three clinical situation gender was a significant variable (Tables 7, 8 and 9). In addition, times of consulting about reproduction problems was another variable that were statistically significant on situation 1 (*P* = 0.028; Table 7), while time spent on solving the problem about reproduction was statistically significant on situation 2 (*P* = 0.020; Table 8). More variables were significant on situation 3, including times of participating in academic forum on breast cancer, times of consulting about reproduction problems, time spent on solving the problem about reproduction (Table 9). In multivariate analysis, significant variables that might influence physicians’ attitudes about whether the patients who need endocrinotherapy still need to continue endocrine treatment after lactation included times of participating in academic forum, times of science popularization about reproduction of early breast cancer, times of consulting about reproduction problems and time spent on solving the problem about reproduction (Table 10). There are four variable that were statistically significant among physicians who would and would not consider recommending patients with breast cancer using of hormone drugs to promote ovulation (Table 11). There are gender, years of practice, times of science popularization about reproduction of early breast cancer and times of consulting about reproduction problems after breast cancer surgery per outpatient service. There are four variables that were significant on physicians’ agreement for a MDT consultation, including gender, times of participating in academic forum on breast cancer, times of science popularization about reproduction of early breast cancer and time spent on solving the problem about reproduction in early breast cancer (Table 12).

**Table 7:** Multivariate analysis of factors that might influence breast cancer-associated physicians’ attitudes about patients with breast cancer in situ getting pregnant after the surgery and radiotherapy. (*N* = 911)

Factor	Propensity, no			OR (95% CI)	Wald *P* value	*P*
	Low	Selective	High			
Gender						.025
Male	272	126	34	1.38 (1.04–1.83)	.025	
Female	328	122	29	Reference		
Come from which area						.050
Developed area	214	75	15	0.74 (0.55–1.00)	.050	
Underdeveloped area	386	173	48	Reference		
Years of practice						.832
< 5 years	280	116	28	0.90 (0.61–1.32)	.578	
5 years ~	186	79	20	0.97 (0.65–1.43)	.861	
10 years ~	134	53	15	Reference		
Volume of breast cancer patients per outpatient service						.173
< 20	303	101	27	0.96 (0.51–1.81)	.904	
20 ~	197	105	23	1.37 (0.74–2.55)	.319	
40 ~	59	28	8	1.24 (0.62–2.49)	.547	
> 60	41	14	5	Reference		
Times of participating in academic forum on breast cancer						.092
< 2	87	50	11	1.67 (0.93–3.00)	.088	
2–4	256	113	22	1.09 (0.66–1.80)	.746	
5–10	191	64	20	0.94 (0.56–1.56)	.804	
≥ 11	66	21	10	Reference		
Times of science popularization about reproduction of early breast cancer						.273
0	199	62	17	0.87 (0.55–1.39)	0.570	
1	168	86	20	1.26 (0.81–1.95)	0.310	
2	117	53	15	1.05 (0.66–1.67)	0.841	
> 2	116	47	11	Reference		
Times of consulting about reproduction problems after breast cancer surgery per outpatient service						.028
1–2	318	100	27	0.80 (0.48–1.35)	.408	
3–4	164	90	20	1.19 (0.71–1.99)	.505	
5–6	53	30	10	1.59 (0.87–2.90)	.128	
> 6	65	28	6	Reference		
Time spent on solving the problem about reproduction in early breast cancer						.638
< 2 min	62	30	5	0.93 (0.52–1.67)	.804	
2 min ~	289	113	27	0.77 (0.50–1.20)	.247	
6 min ~	169	70	21	0.88 (0.56–1.40)	.579	
11 min ~	80	35	10	Reference

**Table 8:** Multivariate analysis of factors that might influence breast cancer-associated physicians’ attitudes about getting pregnant 2 years after surgery in invasive breast cancer patients with lymph nodes-negative. (*N* = 911)

Factor	Propensity, no			OR (95% CI)	Wald *P* value	*P*
	Low	Selective	High			
Gender						< .001
Male	87	206	139	1.83 (1.42–2.37)	< .001	
Female	130	257	92	Reference		
Come from which area						.848
Developed area	67	167	70	0.98 (0.75–1.27)	.848	
Underdeveloped area	150	296	161	Reference		
Years of practice						.903
< 5 years	101	221	102	1.06 (0.75–1.49)	.753	
5 years ~	69	142	74	1.09 (0.76–1.55)	.652	
10 years ~	47	100	55	Reference		
Volume of breast cancer patients per outpatient service						.217
< 20	115	203	113	0.65 (0.37–1.15)	.138	
20 ~	69	176	80	0.82 (0.47–1.43)	.481	
40 ~	23	52	20	0.62 (0.33–1.16)	.133	
> 60	10	32	18	Reference		
Times of participating in academic forum on breast cancer						.788
< 2	37	76	35	0.80 (0.47–1.36)	.405	
2–4	89	212	90	0.87 (0.55–1.37)	.542	
5–10	67	138	70	0.81 (0.52–1.27)	.355	
≥ 11	24	37	36	Reference		
Times of science popularization about reproduction of early breast cancer						.515
0	68	136	74	0.82 (0.54–1.23)	.339	
1	69	139	66	0.80 (0.53–1.18)	.259	
2	45	97	43	0.73 (0.48–1.11)	.141	
> 2	35	91	48	Reference		
Times of consulting about reproduction problems after breast cancer surgery per outpatient service						.103
1–2	113	198	134	1.31 (0.82–2.08)	.259	
3–4	61	167	46	0.94 (0.59–1.50)	.790	
5–6	19	48	26	1.43 (0.82–2.50)	.208	
> 6	24	50	25	Reference		
Time spent on solving the problem about reproduction in early breast cancer						.020
< 2 min	21	45	31	1.59 (0.93–2.71)	.091	
2 min ~	115	216	98	1.05 (0.71–1.55)	.823	
6 min ~	45	145	70	1.55 (1.03–2.36)	.038	
11 min ~	36	57	32	Reference

**Table 9:** Multivariate analysis of factors that might influence breast cancer-associated physicians’ attitudes about getting pregnant 5 years after surgery in invasive breast cancer patients with lymph nodes-positive. (*N* = 911)

Factor	Propensity, no			OR (95% CI)	Wald *P* value	*P*
	Low	Selective	High			
Gender						.003
Male	57	218	157	1.48 (1.14–1.91)	.003	
Female	83	263	133	Reference		
Come from which area						.574
Developed area	44	170	90	0.93 (0.71–1.21)	.574	
Underdeveloped area	96	311	200	Reference		
Years of practice						.185
< 5 years	72	230	122	1.07 (0.75–1.51)	.711	
5 years ~	41	142	102	1.35 (0.94–1.93)	.105	
10 years ~	27	109	66	Reference		
Volume of breast cancer patients per outpatient service						.270
< 20	62	220	149	0.90 (0.51–1.58)	.703	
20 ~	55	176	94	0.75 (0.43–1.32)	.323	
40 ~	15	56	24	0.62 (0.32–1.17)	.138	
> 60	8	29	23	Reference		
Times of participating in academic forum on breast cancer						.005
< 2	28	85	35	0.45 (0.26–0.78)	.004	
2–4	72	210	109	0.57 (0.36–0.90)	.017	
5–10	25	150	100	0.83 (0.52–1.32)	.434	
≥ 11	15	36	46	Reference		
Times of science popularization about reproduction of early breast cancer						.507
0	42	147	89	0.81 (0.53–1.22)	.311	
1	49	145	80	0.77 (0.51–1.15)	.204	
2	31	99	55	0.73 (0.48–1.12)	.154	
> 2	18	90	66	Reference		
Times of consulting about reproduction problems after breast cancer surgery per outpatient service						.034
1–2	64	216	165	1.52 (0.95–2.45)	.081	
3–4	44	167	63	0.97 (0.60–1.56)	.889	
5–6	14	50	29	1.17 (0.66–2.06)	.587	
> 6	18	48	33	Reference		
Time spent on solving the problem about reproduction in early breast cancer						.002
< 2 min	15	51	31	1.19 (0.69–2.04)	.542	
2 min ~	75	242	112	0.90 (0.60–1.34)	.588	
6 min ~	26	131	103	1.65 (1.08–2.52)	.021	
11 min ~	24	57	44	Reference

**Table 10:** Multivariate analysis of factors that might influence breast cancer-associated physicians’ attitudes about whether the patients who need endocrinotherapy still need to continue endocrine treatment after lactation. (*N* = 911)

Factor	Propensity, no			OR (95% CI)	Wald *P* value	*P*
	Low	Selective	High			
Gender						.352
Male	23	141	268	1.14 (0.86–1.51)	.352	
Female	28	176	275	Reference		
Come from which area						.960
Developed area	14	108	182	0.99 (0.74–1.33)	.960	
Underdeveloped area	37	209	361	Reference		
Years of practice						.490
< 5 years	26	165	233	0.99 (0.67–1.44)	.939	
5 years ~	11	97	177	1.19 (0.80–1.77)	.396	
10 years ~	14	55	133	Reference		
Volume of breast cancer patients per outpatient service						.052
< 20	18	136	277	1.28 (0.70–2.35)	.418	
20 ~	23	131	171	0.84 (0.47–1.53)	.573	
40 ~	7	27	61	1.30(0.65–2.58)	.455	
> 60	3	23	34	Reference		
Times of participating in academic forum on breast cancer						< .001
< 2	13	66	69	0.27 (0.15–0.49)	< .001	
2–4	24	146	221	0.51 (0.30–0.86)	.012	
5–10	8	85	182	0.79 (0.46–1.35)	.395	
≥ 11	6	20	71	Reference		
Times of science popularization about reproduction of early breast cancer						.003
0	15	81	182	1.60 (1.02–2.52)	.043	
1	13	90	171	1.63 (1.05–2.53)	.030	
2	19	76	90	0.83 (0.53–1.29)	.396	
> 2	4	70	100	Reference		
Times of consulting about reproduction problems after breast cancer surgery per outpatient service						< .001
1–2	20	116	309	1.46 (0.88–2.42)	.143	
3–4	16	129	129	0.71 (0.43–1.17)	.183	
5–6	10	36	47	0.66 (0.37–1.19)	.165	
> 6	5	36	58	Reference		
Time spent on solving the problem about reproduction in early breast cancer						.047
< 2 min	11	33	53	0.65 (0.36–1.15)	.138	
2 min ~	18	165	246	0.88 (0.57–1.36)	.552	
6 min ~	12	83	165	1.30 (0.81–2.06)	.274	
11 min ~	10	36	79	Reference

**Table 11:** Multivariate analysis of factors that might influence breast cancer-associated physicians’ attitudes about the using of hormone drugs to promote ovulation for patients with breast cancer. (*N* = 911)

Factor	Propensity, no			OR (95% CI)	Wald *P* Value	*P*
	Low	Selective	High			
Gender						< .001
Male	144	216	72	1.64 (1.27–2.13)	< .001	
Female	194	236	49	Reference		
Come from which area						.345
Developed area	108	155	41	1.14 (0.87–1.49)	.345	
Underdeveloped area	230	297	80	Reference		
Years of practice						.039
< 5 years	144	224	56	1.56(1.10–2.21)	.013	
5 years ~	113	130	42	1.25 (0.88–1.80)	.219	
10 years ~	81	98	23	Reference		
Volume of breast cancer patients per outpatient service						.065
< 20	180	201	50	0.69 (0.39–1.22)	.198	
20 ~	110	171	44	0.94 (0.54–1.66)	.842	
40 ~	29	47	19	1.15 (0.61–2.18)	.665	
> 60	19	33	8	Reference		
Times of participating in academic forum on breast cancer						.653
< 2	55	83	10	0.77 (0.45–1.33)	.347	
2–4	151	192	48	0.74 (0.47–1.18)	.203	
5–10	98	139	38	0.80 (0.51–1.27)	.344	
≥ 11	34	38	25	Reference		
Times of science popularization about reproduction of early breast cancer						< .001
0	124	129	25	0.41 (0.27–0.62)	< .001	
1	99	144	31	0.55 (0.37–0.83)	.004	
2	68	91	26	0.50 (0.33–0.77)	.001	
> 2	47	88	39	Reference		
Times of consulting about reproduction problems after breast cancer surgery per outpatient service						.018
1–2	179	212	54	1.43 (0.89–2.30)	.135	
3–4	91	156	27	1.37 (0.85–2.21)	.198	
5–6	28	39	26	2.46 (1.39–4.33)	.002	
> 6	40	45	14	Reference		
Time spent on solving the problem about reproduction in early breast cancer						.708
< 2 min	42	46	9	0.99 (0.57–1.70)	.966	
2 min ~	150	231	48	1.21 (0.81–1.80)	.356	
6 min ~	94	124	42	1.12 (0.74–1.71)	.592	
11 min ~	52	51	22	Reference

**Table 12:** Multivariate analysis of factors that might influence breast cancer-associated physicians’ attitudes about agreement for a MDT consultation to make a full decision on the patients’ reproduction. (*N* = 911)

Factor	Propensity, no			OR (95% CI)	Wald *P* Value	*P*
	Low	Selective	High			
Gender						.027
Male	14	119	299	1.39 (1.04–1.85)	.027	
Female	18	167	294	Reference		
Come from which area						.116
Developed area	4	92	208	1.28 (0.94–1.74)	.116	
Underdeveloped area	28	194	385	Reference		
Years of practice						.463
< 5 years	10	163	251	0.83 (0.56–1.24)	.371	
5 years ~	13	77	195	1.01 (0.66–1.54)	.970	
10 years ~	9	46	147	Reference		
Volume of breast cancer patients per outpatient service						.378
< 20	17	119	295	1.24 (0.66–2.34)	.502	
20 ~	11	115	199	0.93 (0.50–1.74)	.822	
40 ~	3	31	61	0.96 (0.47–1.95)	.909	
> 60	1	21	38	Reference		
Times of participating in academic forum on breast cancer						.001
< 2	10	56	82	0.33 (0.17–0.61)	.001	
2–4	12	133	246	0.53 (0.30–0.92)	.025	
5–10	7	76	192	0.75 (0.43–1.31)	.307	
≥ 11	3	21	73	Reference		
Times of science popularization about reproduction of early breast cancer						< .001
0	12	63	203	2.86 (1.78–4.60)	< .001	
1	10	73	191	2.58 (1.64–4.07)	< .001	
2	5	79	101	1.12 (0.72–1.77)	.611	
> 2	5	71	98	Reference		
Times of consulting about reproduction problems after breast cancer surgery per outpatient service						.062
1–2	14	110	321	1.54 (0.92–2.57)	.098	
3–4	12	106	156	1.03 (0.62–1.71)	.912	
5–6	0	33	60	1.57 (0.85–2.91)	.150	
> 6	6	37	56	Reference		
Time spent on solving the problem about reproduction in early breast cancer						.001
< 2 min	10	31	56	0.35 (0.19–0.66)	.001	
2 min ~	15	143	271	0.57 (0.35–0.91)	.020	
6 min ~	4	80	176	0.92 (0.56–1.51)	.730	
11 min ~	3	32	90	Reference		

## Discussion

We conducted this study to determine breast cancer-associated physicians’ treatment strategies and attitudes regarding reproduction for young patients with early breast cancer with specific clinical problems, and we conducted a multivariate analysis of physicians’ characteristics that might influence their responses to different reproduction problems. To our knowledge, this is the first study to systematically survey breast cancer-associated physicians on this key issue.

In the survey, a total of 911/1249 (72.93%) eligible physicians completed the questionnaire. We identified that male physicians had a more positive attitude toward reproduction, compared with the female physicians. This may be related to the fact that breast cancer patients are more likely to communicate with female physicians than male physicians. We found that 421 (46.2%) physicians with a high propensity believed that reproduction had a definite impact on breast cancer patients and that 439 (48.2%) physicians with a selective propensity stated that reproduction may have an impact on the fetus. This might indicate that higher proportion of physician that reproduction has a greater impact on breast cancer patients. While previous study showing that reproduction did not affect survival in breast cancer patients [10]. Even so, when the patients with early breast cancer ask if she can get pregnant, only 457 (50.2%) physicians in the study thought breast cancer patients may attempt to conceive. This suggests that the problem with physicians recommending reproduction is that they are not very confident. As well as, it was challenging for physicians to decide when may be the proper time for young patients to achieve reproduction based on their clinical situation.

Even though patients with breast cancer in situ may have a lower risk of recurrence, 599 (65.8%) physicians do not recommend immediate reproduction after surgery or radiation, which is in contrast with the 2017 Rehabilitation Therapy Consensus on Breast Cancer in China. While the British Royal Society of Obstetrics and Gynecology recommends that this group of patients may attempt to become pregnant at least 6 months after treatment because of the toxicity of radiotherapy and the need for postoperative endocrinology. For patients with invasive breast cancer, most physicians suggested that patients with negative lymph nodes may attempt reproduction 2 years after surgery, and patients with positive lymph nodes may attempt reproduction 5 years after surgery, which was consistent with the two guidelines above [8, 9]. This suggests that the presence of carcinoma in situ or invasive carcinoma, lymph node metastasis, and postoperative time are factors influencing physicians' decisions regarding reproduction. However, in this study, we did not consider more factors, such as tumor size, pathological grade, invasion of peripheral vascular tumors and status of Her-2 and hormone receptor, into our analyses, which may be a deficiency in our survey.

In this survey, only 177 (19.4%) physicians stated that they might not be influenced by BRCA-1/2 mutation-related patients. At present, the overall survival difference between BRCA-1/2 mutation-related patients who become pregnant and those who do not become pregnant may need to be explored. However, the differences in this issue were not statistically significant among physicians’ demographic characteristics, which indicated that most physicians may take the BRCA-1/2 mutation into consideration to help make decisions for patients.

Previous studies have shown that breastfeeding can reduce the risk of recurrence of breast cancer [11–13], and 329 (36.1%) physicians in this study stated that breast cancer patients may definitely breastfeed after reproduction, which is consistent with the conclusion of the previous study. The British Royal Society of Obstetrics and Gynecology recommends breastfeeding on the unoperated side of the breast because the fibrosis caused by radiotherapy limits galactosis during breast-conserving surgery. Therefore, it’s critical important to develop a practical guide of reproduction planning for young patients with early breast cancer and thus provide more reliable guidelines for the clinical practice.

The advantage of this study is that it is the first clinical research on the reproduction attitude towards the postoperative breast cancer patients. It is a large sample survey of breast cancer-associated physicians in China with a high response rate and that it enables understanding of the attitudes of breast physicians based on specific clinical scenarios. However, there are also some limitations. First, our sample is insufficient, it’s only for Chinese population and lack of international data. Second, when design the questionnaire, we should consider more different scenarios in the same patient in different time from diagnosis and thus be more directly assess the physicians’ recommendations on timing of reproduction. Third, there is no stratification of age stage and no specific investigation into different disciplines, including breast surgeons, breastfeeding consultants, rehabilitation specialists and Radiology Department. Most importantly, we relied on reports from breast physicians about whether they would encourage postoperative pregnancies in patients with breast cancer, and we did not actually have clinical data. In this study, only BRCA-1/2 mutation-related, in situ carcinoma or invasive in situ carcinoma, lymph node metastasis and postoperative time were considered influencing factors for fertility decision-making, and the risk of disease metastasis, recurrence and prognosis of patients should also be systematically evaluated (e.g., tumor size, pathological grade, invasion of peripheral vascular tumors and status of Her-2 and hormone receptor), the lack of which may be a deficiency in our survey. Actually, the key to the decision of reproduction attitude lies in the patients, in the later research, we should focus more on the patients’ attitude.

## Conclusions

This study showed that attitudes towards reproduction of young breast cancer patients from physicians in China. Physicians had a high propensity for recommending reproduction. Compared with both guidelines (2017 Rehabilitation Therapy Consensus on Breast Cancer in China and the British Royal Society of Obstetrics and Gynecology) recommendation when to reproduce in different circumstances for breast cancer patients, physicians from China remained a relatively conservative attitude. Most physicians advised the patients to consult experts from other disciplines, such as gynecology, oncology, genetic and psychology disciplines.
